# The development and validation of the Leiden Bother and Needs Questionnaire for patients with pituitary disease: the LBNQ-Pituitary

**DOI:** 10.1007/s11102-016-0707-4

**Published:** 2016-01-25

**Authors:** Cornelie D. Andela, Margreet Scharloo, Steven Ramondt, Jitske Tiemensma, Olga Husson, Sofia Llahana, Alberto M. Pereira, Ad. A. Kaptein, Noëlle G. A. Kamminga, Nienke R. Biermasz

**Affiliations:** Department of Medicine, Division of Endocrinology and Center for Endocrine Tumors, C7-Q, Leiden University Medical Center, P.O. Box 9600, 2300 RC Leiden, The Netherlands; Medical Psychology, Leiden University Medical Center, Leiden, The Netherlands; Psychological Science, University of California, Merced, CA USA; Department of Medical Psychology, Radboud University Medical Center, Nijmegen, The Netherlands; Center for Behavioural Medicine, University College London, London, UK; Department of Neurology, Leiden University Medical Center, Leiden, The Netherlands

**Keywords:** Patient-reported-outcome, Needs, Quality of life, Illness perceptions, Pituitary adenomas, Acromegaly, Cushing’s disease, Cushing’s syndrome, Prolactinoma, Non-functioning pituitary macroadenoma, Hypopituitarism

## Abstract

**Background:**

Patients report persisting impairment in quality of life (QoL) after treatment for pituitary disease. At present, there is no questionnaire to assess (a) whether patients with pituitary disease are bothered by these consequences, and (b) their needs for support.

**Objective:**

To develop and validate a disease-specific questionnaire for patients with pituitary disease which incorporates patient perceived bother related to the consequences of the disease, and their needs for support.

**Methods:**

Items for the Leiden Bother and Needs Questionnaire for patients with pituitary disease (LBNQ-Pituitary) were formulated based on results of a recent focus group study (n = 49 items). 337 patients completed the LBNQ-Pituitary and six validated QoL questionnaires (EuroQoL-5D, SF-36, MFI-20, HADS, AcroQol, CushingQoL). Construct validity was examined by exploratory factor analysis. Reliabilities of the subscales were calculated with Cronbach’s alphas, and concurrent validity was assessed by calculating Spearman’s correlations between the LBNQ-Pituitary and the other measures.

**Results:**

Factor analyses produced five subscales (i.e., mood problems, negative illness perceptions, issues in sexual functioning, physical and cognitive complaints, issues in social functioning) containing a total of 26 items. All factors were found to be reliable (Cronbach’s alphas all ≥.765), and the correlations between the dimensions of the LBNQ-Pituitary and other questionnaires (all *P* ≤ .0001) demonstrated convergent validity.

**Conclusions:**

The LBNQ-Pituitary can be used to assess the degree to which patients are bothered by the consequences of the pituitary disease, as well as their needs for support. It could also facilitate an efficient assessment of patients’ needs for support in clinical practice. We postulate that paying attention to needs for support will lead to optimal patient care (e.g., improvement in psychosocial care), and positively affect QoL.

**Electronic supplementary material:**

The online version of this article (doi:10.1007/s11102-016-0707-4) contains supplementary material, which is available to authorized users.

## Introduction

Pituitary adenomas can cause several symptoms in the physical, psychological, and social domain, and can be treated by surgery, drug treatment or additional radiotherapy. Symptoms can (partly) resolve upon treatment, but many patients will have permanent hypopituitarism and will require life-long multiple hormone replacement therapy and/or will experience remaining symptoms [1]. In line with these findings, research in patients with pituitary diseases demonstrated that patients report Quality of Life (QoL) impairments [2], also after long-term remission [3–6]. The increasing number of QoL studies in patients with pituitary disease suggests a growing interest in the patient’s perspective [7]. QoL in patients with pituitary disease has been mainly evaluated by generic QoL questionnaires assessing several domains, disease-specific QoL questionnaires assessing disease related QoL aspects, or domain-specific questionnaires assessing particular domain(s) of QoL. Disease-specific QoL questionnaires for pituitary diseases are available for Cushing’s syndrome (i.e., CushingQoL, Tuebing CD-25 [8–10]), acromegaly (AcroQoL [11–13]) and growth-hormone deficiency (QoL-AGHDA [14]), whereas no questionnaires are available for patients with non-functioning pituitary adenoma or prolactinoma.

Recently, we performed a qualitative study utilizing focus group interviews in patients with pituitary diseases in order to further explore the patient’s perspective on QoL [15]. Issues raised in these conversations were compatible with items of available questionnaires, but other topics also emerged. New issues raised that are not covered in existing questionnaires were visual problems, fear of recurrence of the pituitary adenoma, problems with an altered personality, and lack of sympathy and understanding by others. Furthermore, patients reported unmet needs regarding care, such as dissatisfaction with other aspects of medical care i.e., psychological support [15]. In contrast to the large number of studies measuring QoL in patients with pituitary disease, only few studies suggest strategies to improve QoL [7]. Exploration of the patient’s perspective is crucial in identifying potential unmet needs and aspects for improvement in QoL.

Therefore, the aim of the present study was to develop and validate a new questionnaire aiming to assess the degree to which patients are bothered by the consequences of their pituitary disease, as well as their needs for support. The patient’s perspective elucidated during the focus group conversations [15] formed the basis for the development of this questionnaire.

## Patients and methods

### Patients

Patients between 18 and 80 years old with a pituitary disease [i.e., Cushing’s disease (CD), acromegaly (ACRO), prolactinoma (PRL), and non-functioning adenoma (NFA)] monitored at our institute were invited by letter for this study (N = 554). Those who did not respond were contacted by phone and encouraged to participate. A response was received from 408 patients (74 %), but sixty-one of them (15 %) denoted that they did not want to participate. Main reported reasons for not participating were language barrier or perceiving the questionnaire as being too time consuming. Eventually, 347 (63 %) patients completed the questionnaires. Of these, 10 patients filled out <75 % of the LBNQ-Pituitary and were excluded from the analyses, resulting in a total number of 337 (61 %) patients for inclusion. Clinical characteristics of patients were derived from medical records.

### Diagnosis, treatment and follow-up

Details on diagnostic criteria and criteria for remission and follow-up have been previously described: CD [16], ACRO [3], PRL [5], NFA [17]. Essentially, international guidelines for diagnosis, management were followed. At the time of the current study, all patients were in remission or well controlled with medical treatment regimens.

### Procedure

All patients were asked to complete our newly developed questionnaire (see next paragraph), two generic QoL questionnaires and two domain-specific questionnaires. In addition, patients with CD or ACRO were also asked to fill out a disease-specific QoL questionnaire (CushingQoL or AcroQoL, respectively). Based on the preference of the patient, questionnaires were sent by email (online survey) or by regular mail, in order to increase response rate. 255 patients completed the questionnaire online, 82 patients by postal survey. Previous research demonstrated that paper-and-pencil and online surveys did not lead to different results [18]. The Medical Ethical Committee of the LUMC approved this study.

### Development of LBNQ-Pituitary

The items of the Leiden Bother and Needs Questionnaire for patients with Pituitary disease (LBNQ-Pituitary) were derived from recent focus group conversations [15]. The format of the LBNQ-Pituitary was based on the “Belastungsfragebogen Parkinson kurzversion (BELA-P-k)” (Questionnaire on psychosocial Burden and Needs for help in Parkinson’s disease) [19], which has been found to be valid and reliable for Dutch patients with Parkinson’s disease [20].

Consequently, each item consists of three parts. Part A) a screening question to ask whether a certain complaint is present (Yes/To a certain extent/No). For some questions regarding fertility, their family or their partner, patients could also indicate “Not applicable”. Part B) a question on the extent by which the patients is bothered by the complaint (*Bothered by* (Bb)). Part C) a question to assess how much importance patients place on the attention form their healthcare provider for their complaint [*Needs for Support* (NfS)]. Part B and C were scored on a 5-point Likert scale (0 = “not at all” to 4 = “extremely”) and (0 = “not important” to 4 = “extremely important”).

The initial LBNQ-Pituitary consisted of 49 items and one open-ended question (Supplement 1). To establish face validity, items were reviewed by experts from the field i.e., psychologists (MS, NGAK, AAK) and endocrinologists (NRB, AMP). In order to confirm the content and face validity (i.e., relevance, comprehensibility and acceptability of the items), cognitive debriefing interviews with 4 patients were conducted by the investigator (CDA).

### Validated questionnaires to test concurrent validity

#### Generic QOL questionnaires

*EuroQoL*-*5D* (*EQ*-*5D*) assesses the current health status reflected in five health dimensions: mobility, self-care, usual activities, pain/discomfort, and anxiety/depression. Scores are expressed on a 1–3 scale per dimension, with higher scores indicating worse QoL. The questionnaire also includes a visual analogue scale (VAS) ranging from 0 to 100 for recording an individual’s rating of their current health-related well-being, with higher scores indicating a better health status. The EQ-5D was found to be reliable and valid [21].

*MOS Short Form 36* (*SF*-*36*) assesses functional status and general well-being during the previous month. The items cover nine health concepts: (1) physical functioning, (2) social functioning, (3) role limitation (physical), (4) role limitation (emotional), (5) mental health, (6) vitality, (7) pain, (8) general health perception, and (9) general perception of change in health. Scores are expressed on a 0–100 scale, and higher scores indicate a better QoL. The SF-36 has been found to be reliable and valid [22, 23].

#### Domain-specific QoL questionnaires

*Multidimensional Fatigue Inventory* (*MFI*-*20*) assesses fatigue, using a five-point scale. Five different dimensions can be calculated: (1) general fatigue, (2) physical fatigue, (3) reduced activity, (4) reduced motivation, and (5) mental fatigue. Scores vary from 0 to 20; with higher scores indicating greater fatigue. The MFI-20 yields adequate levels of reliability and validity [24].

*Hospital Anxiety and Depression Scale* (*HADS*) assesses anxiety and depressive symptoms and consists of 14 items on a 4-point scale, and both anxiety (7 items) and depression (7 items) scores range from 0 to 21 points. Higher scores indicate more severe anxiety and/or depressive symptoms. A score >8 points on one of the subscales is being used to indicate patients as being anxious or depressed respectively [25]. The HADS yields adequate levels of reliability and validity [26, 27].

#### Disease-specific QoL questionnaires

*AcroQoL* assesses acromegaly-related QoL and consists of 22 questions on a five-point scale. Three different dimensions can be calculated: (1) physical score, (2) psychological-appearance, (3) psychological-personal relations, and a total score. Lower scores indicate worse QoL. The AcroQoL was found to be reliable and valid [11–13].

*CushingQoL* assesses Cushing-related QoL and consists of 12 questions on a five-point scale. The total score ranges from 12 to 60, with a lower score indicating worse QoL. The CushingQoL yields adequate levels of reliability and validity [10, 28].

### Statistics

In order to assess the construct validity of the LBNQ-Pituitary, an exploratory factor analysis was performed on all items using the *Bothered by* (Bb) scores (n = 49). We conducted exploratory factor analysis using oblique rotation. To check for multicollinearity the correlation matrix was studied. The Kaiser–Meyer–Olkin (KMO) measure was used to test for sampling adequacy. KMO can range from 0 to 1, with values near 0 indicating diffusion in the pattern of correlations, and values near 1 indicating compact patterns of correlation. Internal consistency of the LBNQ-Pituitary dimensions was measured using Cronbach’s alpha coefficients.

To establish concurrent validity correlations between Bb scores and scores on the other questionnaires were calculated. Pearson’s correlations were calculated when data were normally distributed and Spearman’s correlations were calculated when data were not normally distributed. Correlation coefficients ranging from .10 to .30 indicate a small effect, .30 to .50 a medium effect, and >.50 a large effect. It was expected that scales that are conceptually related correlate moderately to highly with one another (convergent validity). Conversely, scales with a less clear or absent conceptual relation are expected to show weak correlations (divergent validity). In order to correct for multiple testing the Bonferroni correction was applied and the level of significance was set at *P* ≤ .0001.

Discriminant validity was examined by LBNQ-Pituitary scores between the different pituitary diseases and by using the HADS cut-off points (score >8 points). For the comparison between pituitary diseases an ANOVA was used when data were normally distributed and a Kruskal–Wallis Test was used when data were not normally distributed. For the comparison between patients being clinically anxious or depressed, independent sample t-tests were used when data were normally distributed, and Mann–Whitney U tests when data were not normally distributed. The level of significance was set at *P* < .05.

## Results

### Cognitive debriefing interviews

The LBNQ-Pituitary was completed by four patients in the presence of the investigator (CDA) (3 men and 1 woman; mean age: 57.5 ± 18.7 years). Patients were asked to fill-out the questionnaire and were asked about their thoughts about the questions and whether they thought items were missing. Patients agreed with the items and found it relevant that attention was being paid to the psychosocial consequences of their disease. The LBNQ-Pituitary proved to be feasible and there were no cues for missing items. Only question 49 (‘As a consequence of my pituitary condition, I experience difficulties in performing my work’) was adapted by adding the answer option “Not applicable”.

### Patient characteristics (Table 1)

The full survey was completed by 337 patients (61 % females). The mean age of patients was 56.8 ± 13.7 years with a mean duration since diagnosis of 15.3 ± 11.4 years.

**Table 1:** Patient characteristics

	Total (n = 337)	CD (n = 72)^a^	ACRO (n = 76)	PRL (n = 92)	NFA (n = 97)
Gender (M/F)	131/206	16/56	38/38	23/69	54/43
Age (years)	56.8 (13.7)	54.5 (12.6)	60.6 (13.1)	50.7 (13.3)	61.3 (13.0)
Education [n (%)]					
Low	108 (32 %)	25 (35 %)	33 (43 %)	22 (24 %)	28 (29 %)
Medium	97 (29 %)	20 (28 %)	21 (28 %)	27 (29 %)	29 (30 %)
High	132 (39 %)	27 (37 %)	22 (29 %)	43 (47 %)	40 (41 %)
Marital status [n (%)]					
Single	43 (13 %)	11 (15 %)	7 (9 %)	15 (16 %)	10 (10 %)
Relationship/marriage	262 (78 %)	52 (72 %)	62 (82 %)	68 (75 %)	80 (83 %)
Divorced	17 (5 %)	7 (10 %)	3 (4 %)	5 (5 %)	2 (2 %)
Widow	15 (4 %)	2 (3 %)	4 (5 %)	4 4 %)	5 (5 %)
Pituitary surgery [n (%)]	228 (68 %)	53 (74 %)	68 (90 %)	26 (28 %)	81 (84 %)
Radiotherapy [n (%)]	76 (23 %)	22 (31 %)	19 (25 %)	10 (11 %)	25 (26 %)
Duration of follow-up (years)	15.3 (11.4)	16.2 (13.6)	18.7 (10.6)	16.1 (10.5)	11.3 (10.1)
Medical treatment for the pituitary disease^b^	231 (69 %)	49 (68 %)	52 (68 %)	61 (66 %)	69 (71 %)

*CD* Cushing’s disease, *ACRO* acromegaly, *PRL* prolactinoma, *NFA* non-functioning pituitary adenoma

^a^21 patients were diagnosed with adrenal Cushing’s syndrome, of whom 12 were treated with bilateral adrenalectomy and 10 were treated with unilateral adrenalectomy

^b^Hormonal replacement therapy and/or suppressant medication

### Frequency of reported bother and needs for support (Table 2)

The number of patients who reported to be bothered by a certain complaint (i.e., “This problem and its consequences bother me:” *3. Considerably or 4. Extremely*) were counted, as well as the number of patients who reported a need for support for a certain complaint (i.e., “I find attention from my healthcare providers to be:” *3. Considerably important or 4. Extremely important*). Among the most bothersome complaints, fatigue was mentioned by 63 patients (17 %), while a larger group reported need for support regarding fatigue from their healthcare providers (25 %).

**Table 2:** Top-10 highest bothers and needs for support

↑ Highest bothered by (Bb)	n (%)	↑ Highest needs for support (NfS)	n (%)
Fatigue	63 (17)	Fatigue	84 (25)
Difficulties in performing work	42 (12)	Afraid that pituitary tumour will recur	68 (20)
Problems concentrating	37 (11)	Worried about physical symptoms	65 (19)
More sensitive to stressful situations	35 (10)	Problems concentrating	62 (18)
Pain	35 (10)	Less interested in sex	55 (16)
Going beyond own limits	34 (10)	Mood swings	55 (16)
Less interested in sex	34 (10)	Memory problems	54 (16)
Physical problems during sex	34 (10)	Difficulties in performing work	52 (15)
Sleeping problems	34 (10)	More sensitive to stressful situations	51 (15)
Difficulties letting go of certain thoughts	33 (10)	Sleeping problems	50 (15)

### Construct validity and reliability analysis (Table 3)

Of the initial 49 items, after factor analyses 26 items remained (see Supplement 2 for a detailed description). A factor structure with five factors with eigenvalues over Kaiser’s criterion 1 and a total explained variance of 58.5 % fitted the data best. The KMO measure of sampling adequacy was 0.94 indicating adequate fit for factor analysis (i.e., the data are likely to factor well) [29]. Cronbach alpha’s were calculated for each factor, and all factors were found to be reliable (Cronbach’s alpha .765, or higher).

**Table 3:** Results of final factor analysis existing of 26 items

Item (item no.)	Mood problems	Negative illness perceptions	Issues in sexual functioning	Physical and cognitive complaints	Issues in social functioning
More easily irritated (20)	**.780**	.058	.097	−.074	.034
Changes in personality (18)	**.595**	−.137	.098	−.091	.120
Emotional reactions have changed (19)	**.585**	−.011	.027	−.219	.049
Mood swings (12)	**.584**	−.128	.091	−.125	.022
Anger (23)	**.491**	−.220	−.033	.056	.227
Panic (13)	**.319**	−.078	−.079	−.204	.224
Negative thoughts about how condition will progress (37)	−.028	**−.809**	−.018	−.040	.081
Negative thoughts about the extent to which the condition can be kept under control (38)	−.109	**−.756**	.043	−.029	.163
Negative thoughts about the consequences of the condition (36)	.135	**−.678**	−.050	−.027	.054
Worried about physical symptoms (16)	.218	**−.537**	.070	−.168	−.021
Afraid that pituitary tumour will recur (17)	.240	**−.438**	.159	.089	−.004
Less interested in sex (41)	.010	.040	**.822**	−.063	−.056
Physical problems during sex (40)	−.017	.017	**.783**	.018	.114
Guilt towards partner/close family (26)	.200	−.170	**.305**	−.051	.193
Problems concentrating (6)	.079	.066	.010	**−.766**	.097
Memory problems (8)	.114	.152	−.010	**−.704**	.127
Fatigue (1)	−.023	−.108	.185	**−.694**	−.096
Difficulties in doing several things at the same time (7)	.076	.015	.048	**−.644**	.137
Pain (2)	−.134	−.365	.028	**−.501**	.022
Going beyond own limits (33)	.167	−.135	.052	**−.461**	−.003
Changes in physical appearance (3)	.093	−.174	.052	**−.358**	.036
Circle of friends has become smaller (45)	−.127	−.017	.085	.027	**.847**
Loneliness (25)	.195	−.051	−.087	−.110	**.682**
Feeling uncomfortable in social situations (46)	.058	−.073	.046	.001	**.620**
Lack of understanding of the consequences of the condition from people in social circle (47)	.074	−.028	.025	−.130	**.548**
Feeling the need to be alone (30)	.260	−.038	.089	−.092	**.421**
Cronbach’s alpha	**.889**	**.861**	**.765**	**.876**	**.862**

Factor loadings in bold

α: Cronbach’s alpha coefficient

All items that fell out during factor analyses were inspected (n = 23). Some items appeared to be of interest only for a subset of subjects, for instance, ‘Deteriorated partner relationship’, ‘Worries not being able to have children’ and ‘Feeling to fail in care for family’ and were kept as optional items for these subjects. Furthermore, some items appeared rather disease specific, and of significant interest for the respective diseases; ‘Difficulties letting go of certain thoughts’, ‘Jealousy’, ‘Trouble accepting’, ‘Sleeping problems’, ‘Sadness’ and ‘Shame’ were more relevant to patients with CD, whereas ‘Negative thoughts about medication’ turned out to be more relevant to patients with PRL, and ‘Impaired eyesight’ more relevant to patients with NFA. Therefore, these items (n = 8) were retained in the questionnaire and added as optional questions for patients with CD, PRL or NFA. The sum scores of the subscales were all transformed to a 0–100 scale. The final LBNQ-Pituitary consisted of 26 items, which can be extended by three optional items being relevant for a subset of patients and eight optional items being relevant for a specific pituitary condition. For an overview of retained items see Supplement 3.

### Concurrent validity (Table 4)

As expected, a higher Bb score on Mood problems was strongly associated with worse mood on the EQ-5D, as well as with more anxiety and more depressive symptoms (HADS) (convergent validity). On the other hand, a higher Bb score on Mood problems was also strongly associated with more impairment in social functioning (SF-36) (less divergent validity). Furthermore, in patients with CD a higher Bb on Mood problems was strongly associated with worse disease-specific QoL.

**Table 4:** Significant correlations between *Bothered by* scores on the subscales of the LBNQ-Pituitary and QoL measures

	Mood problems	Negative illness perceptions	Issues in sexual functioning	Physical and cognitive complaints	Issues in social functioning	Total Bb
EQ-5D						
Mobility		.261		.297	.236	.275
Selfcare				.232	.214	.221
Daily activity	.387	.459	.304	**.547**	.449	**.534**
Pain	.302	.369		.480	.337	.421
Mood	.499	.440	.340	.422	.427	**.501**
VAS (well-being)	−.496	−.482	−.335	**−.596**	−.413	**−.599**
SF-36						
Physical functioning	−.358	−.433	−.244	**−.518**	−.418	−.483
Social functioning	**−.599**	**−.534**	−.414	**−.629**	**−.662**	**−.690**
Role limitations physical	−.457	−.489	−.329	**−.639**	**−.530**	**−.611**
Role limitations emotional	−.492	−.406	−.328	**−.569**	**−.531**	**−.561**
Mental health	−.247	−.209				−.220
Vitality	−.252			−.289		−.264
Pain	−.372	−.432	−.240	**−.559**	−.436	**−.505**
General health		−.220		−.257		−.248
Health change						
MFI-20						
General fatigue						
Physical fatigue						
Reduced activity						
Reduced motivation		−.220		−.245	−.220	−.265
Mental fatigue						
HADS						
Anxiety	**.598**	**.552**	.389	**.530**	.471	**.612**
Depression	**.576**	.493	.458	**.632**	**.565**	**.670**
Total score	**.659**	**.572**	.469	**.649**	**.573**	**.716**
CushingQoL	**−.696**	**−.661**	**−.675**	**−.873**	**−.802**	**−.884**
AcroQoL						
Physical score			**−.513**	**−.705**	**−.586**	**−.661**
Psychological-appearance					**−.509**	
Psychological-personal relations			**−.593**		**−.525**	**−.563**
Total score			**−.533**	**−.575**	**−.644**	**−.613**

All Spearman’s correlations, *P* ≤ .0001. Empty cells: correlation was not significant. Bold: correlations (*r* ≥ .500)

A higher Bb score on Negative illness perceptions was strongly associated with more impairment in social functioning (SF-36), more anxiety and a higher total score on the HADS. In patients with CD a higher Bb score on Negative illness perceptions was strongly associated with worse disease-specific QoL.

A higher Bb score on Issues in sexual functioning was associated with more impairment in disease-specific QoL in patients with CD and in patients with ACRO (i.e., AcroQoL, except subscale Psychological appearance).

As expected, a higher Bb score on Physical and Cognitive complaints was strongly correlated with more impairments in the performance of daily activities (EQ-5D), worse general well-being (VAS EQ-5D), more impairments in physical functioning, more physical role limitations, and more pain (SF-36) (convergent validity). On the other hand, a higher Bb score on Physical and Cognitive complaints was also strongly associated with more impairment in social functioning, more emotional role limitations (SF-36), more anxiety and more depressive symptoms (HADS) (less divergent validity). In addition, it was associated with worse disease-specific QoL in patients with CD and in patients with ACRO (i.e., AcroQoL Physical score and Total score) (convergent validity), whereas no significant correlations were found with the AcroQoL subscales Psychological-appearance and Psychological-personal relations (divergent validity).

As expected, a higher Bb score on Issues in social functioning was strongly associated with more impairment in social functioning (SF-36) (convergent validity), whereas also high associations were found with physical and emotional role limitations (SF-36). Furthermore, a higher Bb score on Issues in social functioning was highly associated with more depressive symptoms and a higher total score on the HADS (less divergent validity). In addition, it was associated with worse disease-specific QoL in patients with CD and patients with ACRO (i.e., AcroQoL all subscales).

Finally, a higher total Bb score was strongly associated with more impairment in daily activities, worse mood (EQ-5D), worse general well-being (VAS EQ-5D), more impairment in social functioning, more physical and emotional role limitations, and more pain (SF-36). Likewise, a higher total Bb score was associated with more anxiety and more depressive symptoms (HADS). In addition, a higher Bb total score was associated with worse disease-specific QoL in patients with CD and patients with ACRO (i.e., AcroQoL, except subscale Psychological appearance).

### Discriminant validity

#### Between different pituitary diseases

Patients with CD reported a higher Bb and NfS score on Physical and Cognitive complaints compared to the other groups (ACRO, PRL, NFA) (*P* = .004 and *P* = .043, respectively). Furthermore, patients with CD reported a higher Bb score on Issues in Social functioning, as well as a higher Bb Total score compared to patients with PRL (*P* = .004 and *P* = .023, respectively). In addition, patients with CD reported a higher NfS score on Issues in Social functioning, as well as Total NfS compared to patients with ACRO (*P* = .012 and *P* = .034, respectively) (Supplement 4). On all other subscales of the LNBQ-Pituitary no significant differences were found, pointing to a considerable overlap in perceived consequences between pituitary diseases.

#### Cut-off scores HADS (Fig. 1a, b)

Based on the clinically used cut-off score of the HADS it was observed that 47 patients (14 %) were clinically anxious and 45 (13 %) were clinically depressed. Based on this observation, groups were formed (anxious vs. not anxious; depressed vs. not depressed) and the scores on the Bb subscales of the LBNQ-Pituitary were compared between groups. It was found that patients who could be classified as anxious and/or depressed (>8 points on HADS subscales respectively) showed higher scores on all Bb subscales, as well as the Bb Total score (*P* ≤ .0001).

**Fig. 1:**
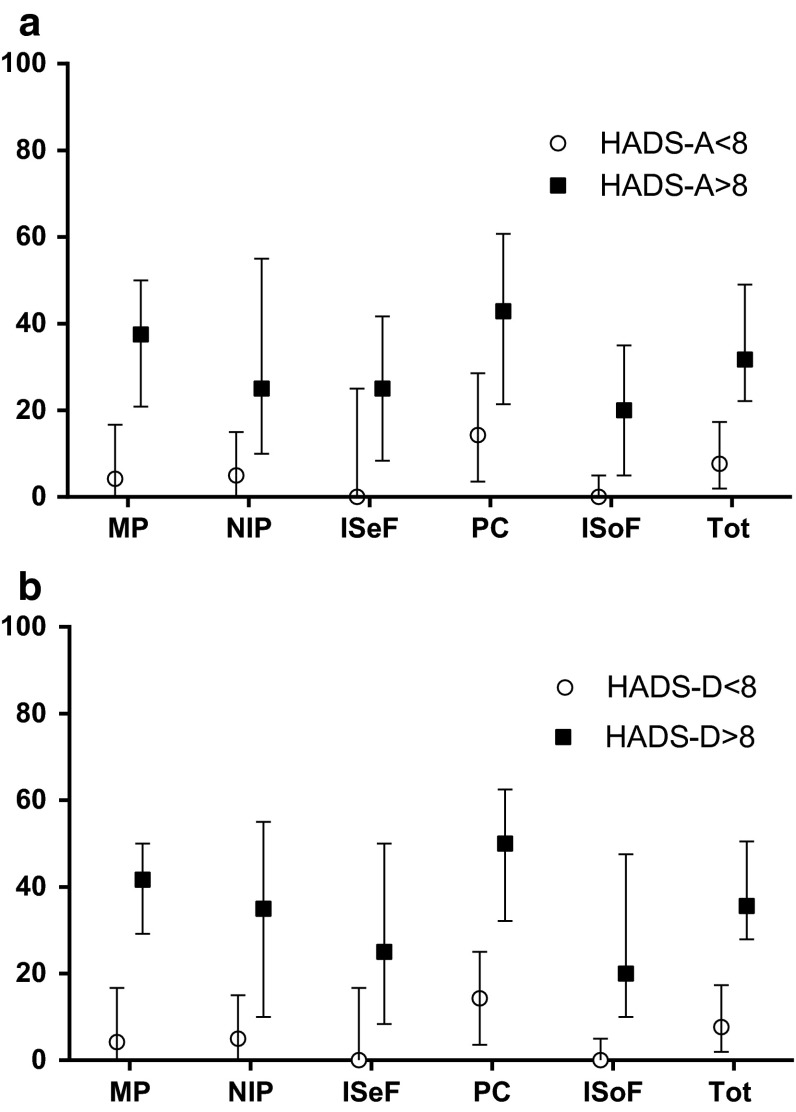
**a** *Bothered by* scores of patients with versus without anxiety. **b** *Bothered by* scores of patients with versus without depression. Median and inter quartile range (IQR). *HADS-A* Anxiety subscale of the Hospital Anxiety and Depression Scale, *HADS-D* Depression subscale of the Hospital Anxiety and Depression Scale, *MP* mood problems, *NIP* negative illness perceptions, *ISeF* issues in sexual functioning, *PC* physical and cognitive complaints, *ISoF* issues in social functioning, *Tot* total score

## Discussion

The present study demonstrated that the resultant factors derived from the exploratory factor analysis of the *Bothered by* (Bb) items of the LBNQ-Pituitary were in accordance with the themes discussed in the focus group conversations i.e., mood problems, negative illness perceptions, issues in sexual functioning, physical and cognitive complaints, and issues in social functioning [15]. Internal consistency of these underlying dimensions was supported by high Cronbach’s alphas. Convergent validity was observed for the subscales Mood problems, Physical and Cognitive complaints and Issues in social functioning. Although divergent validity was also observed by no or weaker correlations with incongruous subscales, some strong correlations were observed between these LBNQ-Pituitary subscales and non-corresponding subscales, such as the strong correlation between Bb subscale Mood problems and Social functioning (SF-36). Furthermore, the LBNQ-Pituitary showed good discriminant validity between patients with various pituitary disease (e.g., patients with CD reported a higher score on Bb and NfS subscales compared to the other groups) and between patients being anxious or depressed as determined by the scores on the HADS.

Based on the results of our recent focus group study [15] it was assumed that physical and cognitive complaints would be identified as two separate dimensions. Surprisingly, in the present study physical complaints and cognitive complaints both loaded on one factor. A possible explanation might be that the question assessing fatigue was not explicitly divided into physical fatigue and mental fatigue. We speculate that specifying this item in future research, might result in fatigue being represented in two factors.

The subscale Negative illness perceptions showed strong correlations with social functioning (SF-36) and anxiety (HADS). These correlations could be explained by previous literature showing that illness perceptions contribute to QoL in patients with pituitary disease [30, 31], and in other patient populations [32, 33]. Furthermore, the subscale Issues in sexual functioning showed strong correlations with disease-specific QoL (i.e., CushingQoL, AcroQoL), whereas only small to moderate associations were found with generic QoL measures. This is probably explained by the fact that both disease-specific QoL measures include items about sexuality, whereas the generic measures do not assess sexuality. This observation points to convergent validity of this subscale. Furthermore, it could be observed that scores on the LBNQ-Pituitary correlate highly with outcomes on the disease-specific questionnaires, which supports the convergent validity in terms of disease specificity.

The observation that strong correlations were observed between incongruous subscales, could possibly be explained by the tight connections between the domains of the biopsychosocial model [34], such as that mood problems might also result in less social functioning. Surprisingly, the LBNQ-Pituitary showed only weak correlations with the Multidimensional Fatigue Inventory-20. This might also be explained by the fact that fatigue was assessed with just one item in the present version of the LBNQ-Pituitary.

Furthermore, the disease-specific bother of pituitary adenomas observed in this study is in accordance with previous literature, with patients with CD reporting the largest negative impact on QoL [7, 35, 36]. The LBNQ-Pituitary offers the possibility to assess bother and needs for support in people with pituitary disease in general with potential comorbid hypopituitarism, while it can also be used to assess aspects related to specific pituitary disease, such as CD or PRL. Moreover, since there are no questionnaires available for patients with NFA or PRL, the LBNQ-Pituitary can be used in these patient groups.

**Fig. 2:**
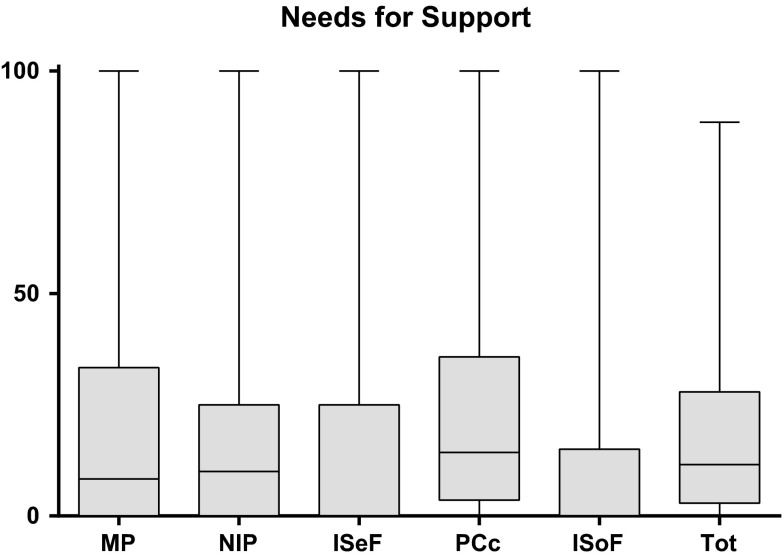
Needs for support. Distribution of needs for support (range 0–100), with a higher score indicating a greater need for support. *MP* mood problems, *NIP* negative illness perceptions, *ISeF* issues in sexual functioning, *PC* physical and cognitive complaints, *ISoF* issues in social functioning, *Tot* total score

To the best of our knowledge, no work has been published reporting a similar questionnaire to the LBNQ-Pituitary which can assess to which extent patients are bothered by consequences of the disease, as well as their needs for support. We postulate that this questionnaire will provide valuable information, in addition to already available QoL data, which is needed for the improvement of psychosocial care in patients with pituitary disease. Furthermore, the LBNQ-Pituitary can be used by clinicians to distinguish between specific bothers and/or specific needs for support. Awareness of patients’ needs for support could facilitate the translation from patients’ needs to optimal patient care. For an overview of the distribution of reported needs for support in our cohort, see Fig. 2. Considering the fact that unmet needs are found to influence QoL [37], and that patients with pituitary disease previously reported unmet needs (e.g., “better cooperation and communication between medical specialties”, “absence of recognition for certain complaints”) [15], we postulate that paying attention to patients’ needs for support will positively affect QoL.

In conclusion, the LBNQ-Pituitary can be used to assess whether patients are bothered by the consequences of the disease, as well as their needs for support. Nevertheless, future research is needed to further establish the psychometric properties, for instance by the use of a confirmatory factory analysis in another cohort in the Netherlands, but also in patients from a different country and with a different language. The LBNQ-Pituitary can be used in clinical research (e.g., to compare bother and needs for support between groups, to evaluate the effect of interventions regarding bother and needs). It can also be used to facilitate the efficient assessment of bother and needs for support in patients with pituitary disease in clinical practice, and further research into this area is warranted.

## Electronic supplementary material

Below is the link to the electronic supplementary material.

Supplementary material 1 (DOCX 47 kb)

Supplementary material 2 (DOCX 14 kb)

Supplementary material 3 (DOCX 16 kb)

Supplementary material 4 (DOCX 15 kb)
