# Supplementary material for: The development and validation of the Leiden Bother and Needs Questionnaire for patients with pituitary disease: the LBNQ-Pituitary
Source: Pituitary. 2016 Jan 25;19:293–302. doi: 10.1007/s11102-016-0707-4 (PMC4858557; doi:10.1007/s11102-016-0707-4)
Supplement: Supplementary file 1 — Supplementary material 1 (DOCX 47 kb) [file 11102_2016_707_MOESM1_ESM.docx]

**Supplement 1a. Format of the LBNQ-Pituitary**

**
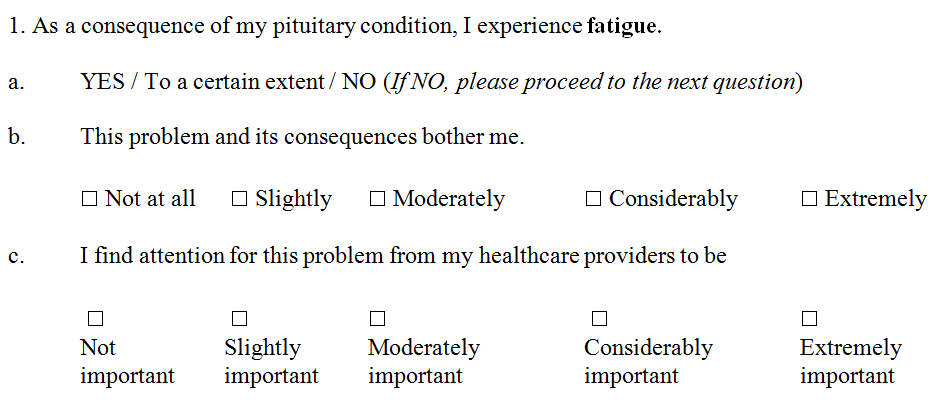
**

**Supplement 1b. Overview of initial items***

1. As a consequence of my pituitary condition, I experience **fatigue.**
2. As a consequence of my pituitary condition, I experience **physical pain.**
3. As a consequence of my pituitary condition, I experience **changes in my physical appearance.**
4. As a consequence of my pituitary condition, I experience **impaired eyesight.**
5. As a consequence of my pituitary condition, I experience **sleeping problems**.
6. As a consequence of my pituitary condition, I experience **problems concentrating**.
7. As a consequence of my pituitary condition, I experience **difficulties in doing several things at the same time.**
8. As a consequence of my pituitary condition, I experience **memory problems**.
9. As a consequence of my pituitary condition, I experience **difficulties letting go of certain thoughts.**
10. As a consequence of my pituitary condition, I **feel down.**
11. As a consequence of my pituitary condition, I experience **anxiety.**
12. As a consequence of my pituitary condition, I experience **mood swings.**
13. As a consequence of my pituitary condition, I have **a tendency to panic in certain situations.**
14. As a consequence of my pituitary condition, I am **more sensitive to stressful situations than before.**
15. As a consequence of my pituitary condition, **I am afraid to faint in certain situations.**
16. As a consequence of my pituitary condition, **I am worried about physical symptoms.**
17. I am **afraid** that the pituitary tumour will recur.
18. As a consequence of my pituitary condition, I experience **changes in my personality.**
19. As a consequence of my pituitary condition, **my emotional reactions have changed.**
20. As a consequence of my pituitary condition, I am **more easily irritated than before.**
21. As a consequence of my pituitary condition, **my confidence has decreased.**
22. As a consequence of my pituitary condition, I experience **shame.**
23. As a consequence of my pituitary condition, I experience **anger.**
24. As a consequence of my pituitary condition, I experience **sadness.**
25. As a consequence of my pituitary condition, I experience **loneliness.**
26. As a consequence of my pituitary condition, I experience **guilt towards my partner/close family.**
27. As a consequence of my pituitary condition, I experience **frustration.**
28. As a consequence of my pituitary condition, **I experience tension.**
29. As a consequence of my pituitary condition, I experience **jealousy towards other (healthy) people.**
30. As a consequence of my pituitary condition, I **sometimes feel the need to be alone for a while.**
31. As a result of my pituitary condition, **I drink more alcohol than previously.**
32. I have **trouble accepting** my pituitary condition and its consequences.
33. I do more than is actually good for me (I go beyond my own limits).
34. I think that every (new) symptom is related to my pituitary condition.
35. I often brood on the **causes** of my pituitary condition.
36. I have negative thoughts about the **consequences** of my pituitary condition.
37. I have negative thoughts about how my pituitary condition will progress.
38. I have negative thoughts about the extent to which my pituitary condition can be kept under **control**.
39. I have **negative thoughts about the medication** I take for my pituitary condition.
40. As a consequence of my pituitary condition, I experience **physical problems during sex.**
41. As a consequence of my pituitary condition, I am **less interested in sex.**
42. As a consequence of my pituitary condition, **I worry that I will not be able to have children.**
43. As a consequence of my pituitary condition, I **feel that I am failing to adequately care for my family.**
44. As a consequence of my pituitary condition, **the relationship with my partner has deteriorated.**
45. As a consequence of my pituitary condition, **my circle of friends has become smaller.**
46. As a consequence of my pituitary condition, **I feel uncomfortable in social situations.**
47. I experience a **lack of understanding of the consequences of my pituitary condition from the people in my social circle.**
48. As a consequence of my pituitary condition, I experience **limitations in engaging in my hobbies.**
49. As a consequence of my pituitary condition, I experience **difficulties in performing my work.**
50. Other problems that I experience: ... (Please also indicate whether you need attention for or coaching in dealing with these problems)

*The Dutch items of the LBNQ-Pituitary were translated by using a forward-backward method i.e., items were first translated into English, and then the English items were translated back into Dutch. Discrepancies were discussed. Then the English items of the LBNQ-Pituitary were presented to seven native English patients being monitored at the department of diabetes and Endocrinology of the University College London Hospital (UK).
