# Supplementary material for: The development and validation of the Leiden Bother and Needs Questionnaire for patients with pituitary disease: the LBNQ-Pituitary
Source: Pituitary. 2016 Jan 25;19:293–302. doi: 10.1007/s11102-016-0707-4 (PMC4858557; doi:10.1007/s11102-016-0707-4)
Supplement: Supplementary file 2 — Supplementary material 2 (DOCX 14 kb) [file 11102_2016_707_MOESM2_ESM.docx]

**Supplement 2. Description of factor analyses**

The first factor analysis was conducted on the initial 49 items. Ten factors had eigenvalues over Kaiser’s criterion of 1 and in combination explained 59.8% of the variance. Items with initial statistics (communalities) <0.30 were excluded (n=8: Difficulties letting go of certain thoughts (9), Negative thoughts about medication (39), Jealousy (29), Limitations in engaging hobbies (48), Deteriorated partner relationship (44), Every (new) symptom being related to condition (34), Sadness (24), Trouble accepting (32)). After excluding these variables, we re-ran the factory analysis.

This second factor analysis was conducted on 41 items. Nine factors had eigenvalues over Kaiser’s criterion of 1 and in combination explained 59.9% of the variance. Items with initial statistics (communalities) <0.30 were excluded (n=4: Feeling down (10), difficulties performing work (49), afraid to faint in certain situations (15), drinking more alcohol than previously (31)). After excluding these variables, we re-ran the factory analysis.

This third factor analysis conducted on the 37 items, indicated eight factors with eigenvalues over Kaiser’s criterion of 1 and in combination explained 60.7% of the variance. Items with initial statistics (communalities) <0.30 were excluded (n=1: Worries not being able to have children (42)). After excluding this variable, we re-ran the factory analysis.

The fourth factor analysis on 36 items indicated seven factors with eigenvalues over Kaiser’s criterion of 1 and in combination explained 60.8% of the variance. Items with initial statistics (communalities) <0.30 were excluded (n=2: Feeling to fail in care for family (43), Impaired eyesight (4)). After excluding these variables, we re-ran the factory analysis.

This fifth factor analysis on 34 items indicated seven factors with eigenvalues over Kaiser’s criterion of 1 and in combination explained 62.7% of the variance. Items which loaded on more than one factor and with differences in factor loadings <.05 were excluded from the analysis (n=2: Frustration (27), Sleeping problems (5)). After excluding these variables, we re-ran the factory analysis.

This sixth factor analysis on 32 items indicated six factors with eigenvalues over Kaiser’s criterion of 1 and in combination explained 61.0% of the variance. Items which loaded on more than one factor and with differences in factor loadings <.05 were excluded from the analysis (n=1: Brood on causes of condition (35)). After excluding this variable, we re-ran the factory analysis.

This seventh factor analysis on 31 items indicated six factors with eigenvalues over Kaiser’s criterion of 1 and in combination explained 61.6% of the variance. Items which loaded on more than one factor and with differences in factor loadings <.05 were excluded from the analysis (n=1: More sensitive to stressful situations than before (14)). After excluding this variable, we re-ran the factory analysis.

This eighth factor analysis on 30 items indicated six factors with eigenvalues over Kaiser’s criterion of 1 and in combination explained 61.6% of the variance. Items which loaded on more than one factor and with differences in factor loadings <.05 were excluded from the analysis (n=1: Shame (22)). After excluding this variable, we re-ran the factory analysis.

This ninth factor analysis on 29 items indicated six factors with eigenvalues over Kaiser’s criterion of 1 and in combination explained 61.1% of the variance. Items which loaded on more than one factor and with differences in factor loadings <.05 were excluded from the analysis (n=1: Tension (28)). After excluding this variable, we re-ran the factory analysis.

This tenth factor analysis on 28 items indicated five factors with eigenvalues over Kaiser’s criterion of 1 and in combination explained 58.3% of the variance. Items which loaded on more than one factor and with differences in factor loadings <.05 were excluded from the analysis (n=1: Confidence has decreased (21)). After excluding this variable, we re-ran the factory analysis.

This eleventh factor analysis on 27 items indicated five factors with eigenvalues over Kaiser’s criterion of 1 and in combination explained 58.2% of the variance. Items which loaded on more than one factor and with differences in factor loadings <.05 were excluded from the analysis (n=1: Anxiety (11)). After excluding this variable, we re-ran the factory analysis.

The twelfth and final factor analysis on 26 items indicated five factors with eigenvalues over Kaiser’s criterion of 1 and in combination explained 58.5% of the variance. Kaiser-Meyer-Olkin (KMO) measure was 0.94 (‘superb’), indicating adequate sample size (29). These five factors were retained in the final analysis. Cronbach’s α were calculated for each factor, and all factors were found to be reliable (> .765).
