# Supplementary material for: The development and validation of the Leiden Bother and Needs Questionnaire for patients with pituitary disease: the LBNQ-Pituitary
Source: Pituitary. 2016 Jan 25;19:293–302. doi: 10.1007/s11102-016-0707-4 (PMC4858557; doi:10.1007/s11102-016-0707-4)
Supplement: Supplementary file 3 — Supplementary material 3 (DOCX 16 kb) [file 11102_2016_707_MOESM3_ESM.docx]

**Supplement 3. Items retained in the LBNQ-Pituitary**

| **Subscale** | **Item (item nr.)** |
| --- | --- |
| 1. **Mood problems** | More easily irritated (20) |
|  | Changes in personality (18) |
|  | Emotional reactions have changed (19) |
|  | Mood swings (12) |
|  | Anger (23) |
|  | Panic (13) |
| 1. **Negative illness perceptions** | Negative thoughts about how condition will progress (37) |
|  | Negative thoughts about the extent to which the condition can be kept under control (38) |
|  | Negative thoughts about the consequences of the condition (36) |
|  | Worried about physical symptoms (16) |
|  | Afraid that pituitary tumour will recur (17) |
| 1. **Issues in sexual functioning** | Less interested in sex (41) |
|  | Physical problems during sex (40) |
|  | Guilt towards partner/close family (26) |
| 1. **Physical & cognitive complaints** | Problems concentrating (6) |
|  | Memory problems (8) |
|  | Fatigue (1) |
|  | Difficulties in doing several things at the same time (7) |
|  | Pain (2) |
|  | Going beyond own limits (33) |
|  | Changes in physical appearance (3) |
| 1. **Issues in social functioning** | Circle of friends has become smaller (45) |
|  | Loneliness (25) |
|  | Feeling uncomfortable in social situations (46) |
|  | Lack of understanding of the consequences of the condition from people in social circle (47) |
|  | Feeling the need to be alone (30) |
| **Relevant for a subset of patients** | Worries not being able to have children (42) |
|  | Feeling to fail in care for family (43) |
|  | Deteriorated partner relationship (44) |
| **Additional items CD** | Difficulties letting go of certain thoughts (9) |
|  | Jealousy (29) |
|  | Troubles with acceptance (32) |
|  | Sleep problems (5) |
|  | Sadness (24) |
|  | Shame (22) |
| **Additional items PRL** | Negative thoughts about medication (39) |
| **Additional items NFA** | Impaired eyesight (4) |
