# Supplementary material for: The development and validation of the Leiden Bother and Needs Questionnaire for patients with pituitary disease: the LBNQ-Pituitary
Source: Pituitary. 2016 Jan 25;19:293–302. doi: 10.1007/s11102-016-0707-4 (PMC4858557; doi:10.1007/s11102-016-0707-4)
Supplement: Supplementary file 4 — Supplementary material 4 (DOCX 15 kb) [file 11102_2016_707_MOESM4_ESM.docx]

**Supplement 4. Bother and Needs for Support between different pituitary adenomas**

|  | **CD**  **(n=72)*** | **ACRO**  **(n=76)** | **PRL**  **(n=92)** | **NFA**  **(n=97)** | ***P* value** |
| --- | --- | --- | --- | --- | --- |
| **Bothered by** |  |  |  |  |  |
| Mood problems | 12.5 (0.0-29.2) | 4.2 (0.0-12.5) | 6.3 (0.0-29.2) | 4.2 (0.0-25.0) | .169 |
| Negative illness perceptions | 10.0 (5.0-20.0) | 5.0 (0.0-15.0) | 5.0 (0.0-18.8) | 5.0 (0.0-20.0) | .118 |
| Issues in sexual functioning | 8.3 (0.0-25.0) | 0.0 (0.0-25.0) | 8.3 (0.0-25.0) | 0.0 (0.0-25.0) | .313 |
| Physical and Cognitive complaints | 25.0 (8.0-46.4)^b, c, d^ | 16.1 (4.5-34.8)^a^ | 14.3 (3.6-25.0)^a^ | 10.7 (3.6-30.4)^a^ | **.004** |
| Issues in Social functioning | 5.0 (0.0-20.0)^b^ | 0.0 (0.0-5.0) | 0.0 (0.0-10.0)^a^ | 0.0 (0.0-5.0) | **.004** |
| Total bothered by | 15.4 (7.7-29.8)^b^ | 7.7 (2.9-17.3) | 10.6 (1.2-19.7)^a^ | 7.7 (2.3-22.1) | **.023** |
| **Needs for Support** |  |  |  |  |  |
| Mood problems | 12.5 (0.0-41.7) | 4.2 (0.0-16.7) | 8.3 (0.0-36.5) | 8.3 (0.0-29.2) | .163 |
| Negative illness perceptions | 15.0 (5.0-25.0) | 10.0 (0.0-20.0) | 10.0 (0.0-23.8) | 10.0 (0.0-30.0) | .072 |
| Issues in sexual functioning | 8.3 (0.0-25.0) | 0.0 (0.0-25.0) | 0.0 (0.0-33.3) | 0.0 (0.0-25.0) | .364 |
| Physical and Cognitive complaints | 25.0 (4.5-56.3)^b, c, d^ | 14.3 (3.6-31.3)^a^ | 14.3 (0.0-32.1)^a^ | 14.3 (0.0-32.1)^a^ | **.043** |
| Issues in Social functioning | 5.0 (0.0-23.8)^d^ | 0.0 (0.0-5.0)^a^ | 0.0 (0.0-10.0) | 0.0 (0.0-10.0) | **.012** |
| Total Needs for Support | 20.2 (6.0-32.5)^d^ | 8.7 (1.9-19.2)^a^ | 11.1 (1.0-27.9) | 8.7 (3.4-24.5) | **.034** |

Data is presented as median and inter quartile range (IQR). Non-parametric Kruskal Wallis Test, P<.05.

*21 patients were diagnosed with adrenal Cushing’s syndrome, of whom 12 were treated with bilateral adrenalectomy and 10 were treated with unilateral adrenalectomy. CD: Cushing’s disease; ACRO: acromegaly; PRL: prolactinoma; NFA: non-functioning pituitary adenoma. ^a^compared to CD, ^b^compared to PRL, ^c^compared to NFA, ^d^compared to ACRO.
